# Effect of NOciception Level-Directed analgesic management on Opioid usage in Robot-assisted laparoscopic radical prostatectomy (NOLDOR): study protocol for a single-centre single-blinded randomised controlled trial

**DOI:** 10.1016/j.bjao.2022.100112

**Published:** 2022-11-21

**Authors:** Yuma Kadoya, Nobuhiro Tanaka, Takanori Suzuka, Takayuki Yamanaka, Mitsuru Ida, Yusuke Naito, Shota Suzuki, Shu Kasama, Naoki Ozu, Masahiko Kawaguchi

**Affiliations:** 1 Department of Anaesthesiology, Nara Medical University, Kashihara, Nara, Japan; 2 Institute for Clinical and Translational Science, Nara Medical University Hospital, Kashihara, Nara, Japan

**Keywords:** inflammatory biomarker, nociception level-guided anaesthesia, nociception monitor, opioid consumption, robot-assisted laparoscopic radical prostatectomy

## Abstract

**Background:**

The nociception level (NOL) index discriminates noxious stimuli during surgery with high sensitivity and specificity. Although some studies have reported that a NOL-directed opioid protocol reduces intraoperative opioid consumption, one study implied that it might cause an unintended increase in the stress response. Therefore, we designed a study to investigate the effects of the NOL-directed opioid protocol and measure inflammatory biomarkers.

**Methods:**

This single-centre RCT will enrol 54 patients undergoing robot-assisted laparoscopic radical prostatectomy. Eligible patients will be randomly allocated to receive (i) NOL-directed intraoperative opioid management (NOL group) or (ii) conventional intraoperative analgesic management (control group). The remifentanil infusion rate will be determined solely using the NOL index during surgery in the NOL group. The primary outcome will be the mean intraoperative remifentanil infusion rate. Secondary outcomes will include the plasma concentrations of three perioperative inflammatory biomarkers (interleukin-6, C-reactive protein, and cortisol) and the variation in the NOL index at the start of pneumoperitoneum and with postural changes.

**Conclusions:**

This study is expected to accumulate evidence on the effects of NOL-directed analgesic opioid protocol and provide additional evidence regarding the variability of stress responses and the character of the NOL index.

**Clinical trial registration:**

JRCTs052220034.

The dosing of potent opioids during anaesthesia depends on the anticipated degree of noxious stimuli, with adjustments made in response to variation in the heart rate and blood pressure and the stage of surgery. Provision of optimal intraoperative analgesia is therefore dependent on the skill and experience of the anaesthesiologist. Remifentanil, which is used commonly during surgery, can lead to postoperative opioid-induced hyperalgesia if used at higher doses,^1^ resulting in sympathomimetic stimulation and increased postoperative pain scores.^2^ Thus, dose titration of remifentanil is of utmost importance.

The PMD-200™ monitoring device (Medasense Biometrics Ltd, Ramat Gan, Israel) provides the nociception level (NOL) index.^3^ Previous studies have shown that NOL-guided opioid administration decreases the remifentanil dose,^4^ postoperative pain scores,^4^^,^^5^ and episodes of hypotension.^5^ However, one study reported an increased stress response in the group using the NOL index associated with a decrease in intraoperative remifentanil consumption.^4^ The authors implied that an average dose of remifentanil below 0.2 μg kg^−1^ min^−1^ might lead to an unintended increase in intraoperative stress response. Based on this result, the threshold for the infusion rate of remifentanil under NOL-directed opioid management needs to be determined.

Additionally, an evaluation of perioperative inflammatory biomarkers is important, as the aforementioned study implied that NOL-directed opioid management might cause unintended stress responses.^4^ Although previous studies^4^^,^^5^ evaluated cortisol and adrenocorticotropic hormone levels as perioperative inflammatory biomarkers, a systematic review^6^ showed that concentrations of interleukin-6 (IL-6) and C-reactive protein (CRP) are more closely associated with surgical invasiveness than cortisol concentration. Thus, we hypothesised that, amongst patients undergoing similar surgery, concentrations of IL-6 and CRP would be influenced by the analgesic protocol to a greater extent than the cortisol concentration. Further, setting a minimum infusion rate for remifentanil (e.g. 0.1 μg kg^−1^ min^−1^) could prevent elevations in inflammatory biomarker levels. In addition, although pneumoperitoneum and postural changes may affect the NOL index, variations during these interventions have not been evaluated.

Therefore, we designed a single-centre single-blinded RCT to investigate the impact of a NOL-directed opioid administration protocol on intraoperative remifentanil consumption, measuring inflammatory biomarker levels to investigate unintended stress responses. Eligible patients will be randomly allocated to receive NOL-directed intraoperative opioid management (NOL group) or conventional intraoperative analgesic management (control group), with a 1:1 allocation with block randomisation.

## Methods

### Study setting

This study will be performed at Nara Medical University Hospital in Nara, Japan.

### Eligibility criteria

**Fig 1:**
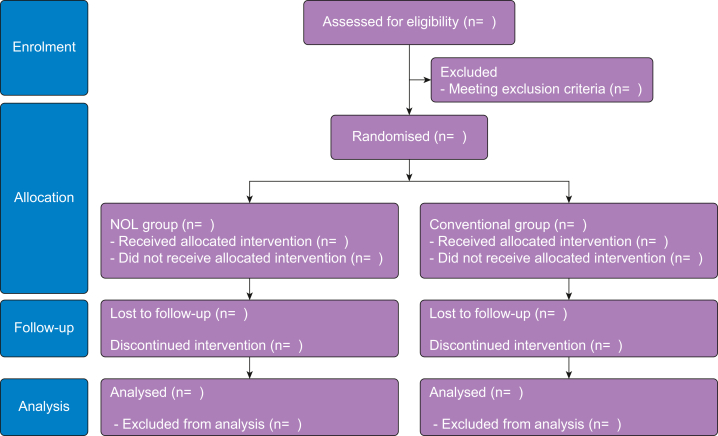
NOciception Level-Directed analgesic management on Opioid usage in Robot-assisted laparoscopic radical prostatectomy (NOLDOR) flow diagram. NOL, nociception level.

Male patients (aged 20–85 yr) classified as ASA physical status 1–3, scheduled for robot-assisted laparoscopic radical prostatectomy (RARP), and who provide written informed consent will be eligible for inclusion. The exclusion criteria are as follows: emergency surgery, treatment with beta blockers, oral steroid use, no suitable finger for measurements, or inability to provide consent. Figure 1 shows the patient flow diagram.

### Intervention

#### NOL index

The NOL index reflects the nociceptive state and is measured using four sensors incorporated on the finger probe of the PMD-200: peripheral temperature, galvanic skin response, accelerometer, and photoplethysmography.^3^ The index ranges from 0 (complete absence of nociceptive response) to 100 (extreme nociceptive response). The manufacturer suggests that an index between 10 and 25 represents sufficient analgesia, and this threshold has been used in multiple trials.^4^^,^^5^^,^^7^

### Study procedures and anaesthetic management

#### Both groups

A finger probe to measure the NOL index located contralateral to a noninvasive blood pressure cuff will be attached before the induction of anaesthesia. The 3M™ Bair Hugger™ warming blanket system (3M, St. Paul, Minnesota, USA) on the upper body will be used for warming. Anaesthesia will be maintained with sevoflurane 1–1.5% and rocuronium, guided by train-of-four (TOF) neuromuscular responses to induce deep muscle relaxation throughout the surgery. Although several anaesthetic factors can affect the levels of inflammatory biomarkers, the type of anaesthetic agent used does not affect the concentrations of the inflammatory biomarkers selected for this study.^8^^,^^9^

Fluid management will be at the discretion of the anaesthesiologist. When the mean arterial blood pressure falls below 60 mm Hg, a bolus of vasopressor (phenylephrine, ephedrine, norepinephrine) or an infusion of phenylephrine or norepinephrine will be administered. This threshold can be varied for patients with cardiovascular disease at the discretion of the anaesthesiologist. Ondansetron 4 mg and acetaminophen 1000 mg or 15 mg kg^−1^ (if body weight <50 kg) will be administered intravenously at the end of surgery. Dexamethasone will be excluded to avoid confounding the inflammatory marker responses. The patient's trachea will be extubated only when the TOF ratio is 100%, with sufficient reversal using sugammadex. After terminating anaesthesia, patients will receive i.v. fentanyl patient-controlled analgesia by CADD®-Solis PIB (Smiths Medical, St Paul, MN, USA), with fentanyl concentration 0.5 μg kg^−1^ min^−1^, bolus 1 ml on demand, and a 10 min lock-out interval. After the surgery, patients will receive acetaminophen (every 6 h for 24 h and thereafter on demand) and NSAIDs on demand. Intraoperative beta blocker or steroid administration during surgery is prohibited.

#### Control group

The NOL index on the PMD-200 will be concealed from the anaesthesiologist in charge. Remifentanil and fentanyl will be administered to achieve analgesia; the dose will be controlled by the anaesthesiologist monitoring the patient's vital signs and procedure.

#### NOL group

To determine the baseline remifentanil infusion rate and the dose of fentanyl, we investigated conventional remifentanil and fentanyl consumption during RARP at our hospital over the last 2 yr. The mean remifentanil dose was approximately 0.18 μg kg^−1^ min^−1^. Tracheal intubation and skin incisions require higher remifentanil doses. Thus, we set the baseline remifentanil infusion rate as 0.2 μg kg^−1^ min^−1^, followed by a bolus of remifentanil 30 μg. The mean fentanyl dose was approximately 350 μg; this study was designed to have a similar mean dose.

**Fig 2:**
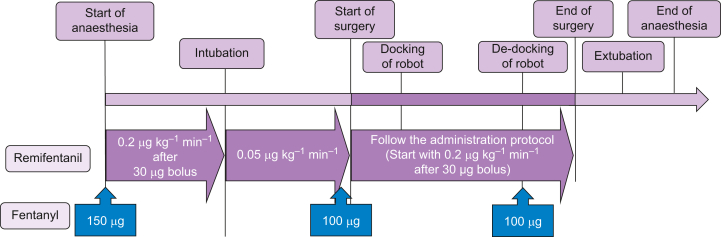
NOciception Level-Directed analgesic management on Opioid usage in Robot-assisted laparoscopic radical prostatectomy (NOLDOR) study intraoperative flowchart for the NOL group. NOL, nociception level.

After 3 min of continuous baseline remifentanil infusion, propofol 0.5–2 mg kg^−1^, fentanyl 150 μg, and rocuronium 0.6–1.2 mg kg^−1^ will be administered. After tracheal intubation, the remifentanil infusion rate will be decreased to 0.05 μg kg^−1^ min^−1^. The rate will be increased to 0.2 μg kg^−1^ min^−1^ before the skin incision, followed by a bolus of remifentanil 30 μg and fentanyl 100 μg. Thereafter, the remifentanil dose will be determined according to the administration protocol. An additional fentanyl 100 μg will be administered for postoperative pain around the time of robot de-docking. Figure 2 shows the perioperative flowchart.

In the NOL group, the remifentanil infusion rate will be determined using the NOL index during surgery. The remifentanil infusion rate will be reduced by 0.03 μg kg^−1^ min^−1^ when the NOL index is under 10 for more than 30 s and increased by 0.03 μg kg^−1^ min^−1^ when the NOL index is over 25 for more than 30 s, followed by a bolus of remifentanil 30 μg. Because a rate of less than 0.1–0.2 μg kg^−1^ min^−1^ can elevate stress hormone levels, an infusion rate above 0.11 μg kg^−1^ min^−1^ will be maintained.^4^ The NOL index will be reassessed after 5 min when changing the infusion rate (Table 1). If the NOL index increases unexpectedly during the 5 min interval and the anaesthesiologist in charge considers it as potentially harmful for the patient, they will consider deviating from the opioid protocol to reassess it. The investigators will instruct the anaesthesiologists and provide explanation forms for each patient to improve adherence to the intervention protocol.

**Table 1:** Administration protocol for remifentanil. NOL, nociception level.

NOL	Remifentanil infusion rate	
NOL <10 for more than 30 s	Decreased by 0.03 μg kg^−1^ min^−1^ (maintained over 0.11 μg kg^−1^ min^−1^)	Next assessment is 5 min later
10≤ NOL ≤25	Maintained	
NOL >25 for more than 30 s	Bolus 30 μg and increased by 0.03 μg kg^−1^ min^−1^	Next assessment is 5 min later

#### Surgical procedure

All patients will undergo RARP in the 25° Trendelenburg position using the AirSeal® insufflation system (SurgiQuest, Milford, CT, USA), with a low pressure of 8–10 mm Hg.

#### Criteria for discontinuing interventions

The investigators will discontinue allocated interventions for the following reasons: (i) participants withdraw informed consent, (ii) participants become ineligible after registration, (iii) change of planned surgical procedure, (iv) severe complications occurring during surgery (e.g. massive bleeding or anaphylactic shock), (v) trial discontinuation, and (vi) other reasons.

### Primary and secondary outcomes

The primary outcome is the mean intraoperative remifentanil infusion rate per ideal body weight (IBW). The infusion rate will be calculated as total amount of intraoperative remifentanil divided by the duration of the surgery, and IBW will be calculated as 22 × (height in metres).^2^

Plasma inflammatory biomarker concentrations, including IL-6 and CRP, will be compared between the groups as secondary outcomes. The time points for the assessment of these biomarkers were selected based on previous studies,^6^^,^^8^ which showed that IL-6 concentrations increased 0–2 h postoperatively and peaked at 12–24 h postoperatively, whereas CRP concentrations showed a delayed peak at 1–3 days postoperatively. As in previous studies,^3^^,^^6^ we will measure cortisol concentrations at baseline and at the end of surgery.

Variations in the NOL index at the start of pneumoperitoneum and in response to postural changes will be investigated as secondary outcomes. These sub-analyses will provide information on the clinical limitations of anaesthetic management using the NOL index.

The secondary outcomes include the following: (i) perioperative IL-6, CRP, and cortisol concentrations (IL-6 and cortisol will be assessed by electrochemiluminescence immunoassay); (ii) total remifentanil dose; (iii) variation from baseline in the NOL index 60–90 s after each of tracheal intubation, the onset of pneumoperitoneum, and any postural changes; (iv) postoperative pain score (numeric rating scale [NRS]) 2 h after surgery and on postoperative Days 1, 2, 3, and 7; (v) incidence of postoperative nausea and vomiting (PONV) 2 h after surgery and on postoperative Days 1, 2, and 3; (vi) dose of postoperative opioid fentanyl and analgesic agents; (vii) occurrence of chronic pain at 3 months postoperatively; (viii) dose of vasoactive agents (phenylephrine, ephedrine, norepinephrine, and atropine); (ix) duration of hypotension (MAP below 60 mm Hg), hypertension (systolic BP over 160 mm Hg), bradycardia (heart rate less than 40 beats min^−1^), and tachycardia (heart rate more than 100 beats min^−1^).

**Fig 3:**
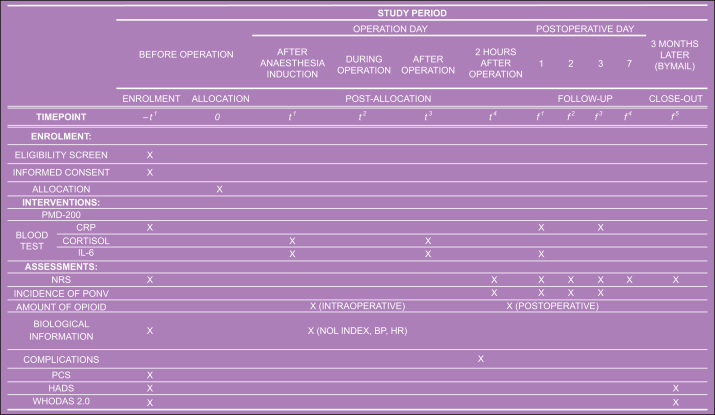
Assessment schedule. BP, blood pressure; CRP, C-reactive protein; HADS, Hospital Anxiety and Depression Scale; HR, heart rate; IL-6, interleukin-6; NOL, nociception level; NRS, numeric rating scale; PCS, Pain Catastrophizing Scale; PONV, postoperative nausea and vomiting; WHODAS 2.0, WHO Disability Assessment Schedule 2.0.

Several blood samples will be collected to evaluate the concentrations of the biomarkers (Fig. 3). The time points of blood sampling, including routine care for the procedure, are several days before surgery (–*t*^1^), after induction of anaesthesia (*t*^1^), after the procedure (*t*^3^), on postoperative Day 1 (*f*^1^), and on postoperative Day 3 (*f*^3^). IL-6 will be measured at *t*^1^, *t*^3^, and *f*^1^; CRP at –*t*^1^, *f*^1^, and *f*^3^; and cortisol at *t*^1^ and *t*^3^.

To evaluate chronic post-surgical pain, we will use the Japanese versions of three questionnaires in addition to the NRS: Pain Catastrophizing Scale (PCS),^10^ Hospital Anxiety and Depression Scale (HADS),^11^ and WHO Disability Assessment Schedule 2.0 (WHODAS 2.0).^12^ Patients will complete the three questionnaires on the day before surgery, and the HADS and WHODAS 2.0 at 3 months postoperatively by mail (Fig. 3).

The nociceptive response (NR), a recently proposed new concept that quantifies nociceptive stimuli but has not yet been fully applied clinically, will be measured as an exploratory outcome to examine the relationship between NR and NOL. The NR is calculated as –1+2/(1+exp[–0.01 HR–0.02 SBP+0.17 PI]), where HR is heart rate, SBP is systolic blood pressure and PI is the perfusion index.^13^

### Sample size calculation

The sample size was calculated based on a previous meta-analysis^14^ that found NOL-guided analgesia protocols to decrease intraoperative remifentanil consumption (standardised mean difference [SMD] –0.68; 95% confidence interval: –1.13 to 0.24; *P*=0.003). We will focus on one disease and procedure and the NOL index alone in the present trial, and thus, we expect less variability. Accordingly, we estimated SMD as 0.8 to obtain group sample sizes of 25 patients achieving 80% power with a significance level of 0.05. Considering a dropout rate of 10%, we will enrol 27 patients in each group.

#### Recruitment

All patients scheduled for RARP will be recruited at Nara Medical University. The investigators will select eligible participants and provide an explanation regarding this study.

#### Allocation

Participants will be randomly assigned to each group with a 1:1 allocation using a web-based service (QuickCalcs; GraphPad Software, La Jolla, CA, USA). The block sizes will not be disclosed to ensure concealment. Randomisation will not be stratified, as there is little possibility of imbalance between each group because we selected only one procedure.

Blinding is only possible for the participants. The anaesthesiologists will open a sealed envelope containing the group name before the operation. The allocation manager will perform randomisation. Participants will be unblinded after 3 months of follow-up, if requested.

#### Data collection methods

Consumption of opioid and other agents, NOL index, information during surgery, and complications will be collected from electronic medical records and transferred to paper case report forms. Blood tests for IL-6, CRP, and cortisol will be taken at designated points. NRS and PONV will be assessed through face-to-face interviews. Patients will complete three questionnaires (PCS, HADS, and WHODAS 2.0) on the day before the surgery. The HADS, WHODAS 2.0, and NRS questionnaires will be sent to participants by mail 3 months later and sent back to the investigators after completion.

#### Data management and monitoring

All collected data will be stored anonymously and secured at the data centre of Nara Medical University. Only investigators will have access to the data; however, access will be permitted for audits, monitoring, and inspections by concerned authorities. Data monitoring will be performed by a data monitoring committee comprising designated staff who are not involved in the study to ensure accuracy and completion. All collected data will be monitored after the first case and after every 10 cases subsequently. The results will be reported to the investigators in writing, and the investigators will make the final decision regarding trial termination. We do not expect interim analyses, as the possibility of severe adverse events is extremely low.

#### Statistical methods

Baseline characteristics will be summarised as summary statistics (min, median, and max) for continuous variables and as frequency and proportion for binary variables.

For the primary outcome, the difference between the groups will be examined using an unpaired *t*-test. The mean value and its 95% confidence interval of the difference between the groups will be obtained.

Secondary outcomes (i), (ii), (v), (vii), and (viii) will be analysed as follows: the mean and its 95% confidence interval for each time point and group will be estimated. The mean and its 95% confidence interval for the difference between the groups at each time point will be estimated. Secondary outcomes (iv) and (vi) will be analysed as follows: the proportion and its 95% confidence interval for each time point and group will be estimated. The mean and its 95% confidence interval for the difference between the groups at each time point will be estimated. This procedure will be repeated for the cumulative incidence rate using the Kaplan–Meier method, if necessary.

Exploratory analysis of the relationship between NOL and NR will mainly be performed using correlation coefficients and some plots.

### Harms

The intervention is wearing a finger probe similar to a pulse oximetry probe, which is not associated with complications.

We will take intraoperative blood samples from the arterial line, which is routinely placed during surgery, and take postoperative blood samples during the course of routine postoperative care. As the additional volume of collected blood is minimal, it will not affect the condition of the patients.

The NOL-guided protocol may cause excessive or insufficient analgesia because the remifentanil infusion rate will be determined solely by the NOL in the intervention group. However, meta-analyses reported that nociception monitoring does not increase intraoperative opioid consumption,^14^, ^15^, ^16^ and we will avoid insufficient analgesia by maintaining a remifentanil infusion rate >0.1 μg kg^−1^ min^−1^.^4^

## Dissemination plans

Dissemination plans include presentations in peer-reviewed scientific journals and at international conferences.

### Ethics and dissemination

#### Ethics approval

The study protocol was approved by the Certified Review Board of Nara Medical University on May 11, 2022 (CRB5200002). This study will be conducted in accordance with the Declaration of Helsinki and adhere to the Clinical Trial Act. Written informed consent will be obtained from all individual participants included in this study by investigators. No individual person's data will be used for this study.

### Protocol amendments

If the study protocol requires amendment, all of the content of the revision and its reasons will be reported to the institutional ethics committee. After the re-examination and approval, the investigators will revise the instructions for patients.

### Ancillary and post-trial care

There is no post-trial care in this protocol. The investigator will have insurance to compensate the participants for any harm related to this trial.

## Funding

Japan Society for the Promotion of Science (JP 22K16606); Nara Medical University Clinical Research Grant Program (21-003).

## Authors' contributions

Study conception: NT

Study protocol design: YK, NT, SS, SK

Obtaining of funds: NT, MK

Development of protocol: TS, TY, MI, YN, MK

Provision of information for proper operations: SS, SK

Data collection: YK, NT, TS, TY

Data acquisition: TS, TY, MI, YN, MK

Statistical expertise for the protocol: NO

Drafting of article: YK

Critical review of protocol: all authors

Substantial revision of article: all authors

Approval of final article: all authors
